# Experiences of family caregivers of patients with terminal disease and the quality of end-of-life care received: a mixed methods study

**DOI:** 10.7717/peerj.10516

**Published:** 2020-12-14

**Authors:** Celia Martí-García, Manuel Fernández-Alcántara, Patricia Suárez López, Carolina Romero Ruiz, Rocío Muñoz Martín, Mᵃ Paz Garcia-Caro

**Affiliations:** 1 Department of Nursing, University of Málaga, Málaga, Spain; 2 Mind, Brain and Behaviour Research Center (CIMCYC), University of Granada, Granada, Spain; 3 Department of Health Psychology, University of Alicante, Alicante, Spain; 4 Distrito sanitario Granada-Metropolitano de Atención Primaria, Granada, Spain; 5 Hospital General Universitario Virgen de las Nieves, Granada, Spain; 6 Department of Nursing, University of Granada, Granada, Spain

**Keywords:** Palliative care, Qualitative, Mixed methods, Bereavement, Relatives, End-of-life care, Caregiver

## Abstract

The aim of this study was to analyze the perceptions and experiences of relatives of patients dying from a terminal disease with regard to the care they received during the dying process, considering the oncological or non-oncological nature of the terminal disease, and the place where care was provided (at home, emergency department, hospital room, or palliative care unit). For this purpose, we conducted a mixed-methods observational study in which two studies were triangulated, one qualitative using semi-structured interviews (*n* = 30) and the other quantitative, using questionnaires (*n* = 129). The results showed that the perception of relatives on the quality of care was highly positive in the quantitative evaluation but more critical and negative in the qualitative interview. Experience of the support received and palliative measures was more positive for patients attended in hospital in the case of oncological patients but more positive for those attended at home in the case of non-oncological patients.

## Introduction

The purpose of care in the advanced or terminal stage of a disease is to promote the adaptation of patients and relatives to the new reality (Rodrigues-Gomes, 2010). The proximity of death generates suffering in both patients and relatives, increasing the high demand for care in the terminal stage (Moreirade Souza & Turrini, 2011). Quality end-of-life care involves healthcare professionals, patients, and caregivers, while family members offer an important perspective on their experience of the disease and the care provided (Mori et al., 2012).

High-quality end-of-life care include physical comfort, emotional support for patients, the coordination of care in the different care settings, and the provision by healthcare professionals of adequate information and emotional support to relatives (Teno et al., 2004). Satisfaction with care is defined by the difference between expectations and perceptions, and perceptions are generally considered a strong predictor of satisfaction (Rhodes et al., 2008).

There appear to be many gaps in research on the satisfaction of patients and relatives with end-of-life care. In the case of family members, most studies have used instruments designed for patients, which can result in a biased interpretation (Almaraz, Aldasoro & Sobradillo, 2006; Jongerden et al., 2013). The stress experienced by relatives of terminal patients is distinct from that of the patients, and their evaluation may be influenced by their own experience of loss, among other factors, including the relationships and communications among family members (Toscano, Bernabeu & Ollero, 2010). Experience of the end-of-life process also appears to differ between relatives of patients with an oncological versus non-oncological disease. There is a greater recognition of the association of death with oncological diseases, for which palliative care resources are more highly developed and more widely implemented (Gómez-Batiste & Calsina-Berna, 2015; Lunney et al., 2003). In addition, a recent retrospective study observed that healthcare professionals appear to find the identification of terminal situations more difficult in non-oncological than oncological patients (Campos-Calderón et al., 2017). Although palliative care has been integrated into the Spanish health system, specialized palliative care is still mainly delivered to cancer patients. Various barriers have been identified that hamper its provision to other groups of patients with complex problems and a high need for palliative care, including a lack of clarity about the prognosis, a strong emphasis on a curative approach, and a reluctance to talk about death (Penders et al., 2017; Guardia-Mancilla et al., 2017; Guardia Mancilla et al., 2018).

The activity of healthcare professionals and the experience of patients and relatives differ between hospital and home settings (Gallagher & Krawczyk, 2013; Teno et al., 2004), and end-of-life care is known to vary among hospital departments (e.g., internal medicine and intensive care), although a markedly lower variability has been observed among professionals in palliative care, oncology, and primary care (Guardia-Mancilla et al., 2017).

Both variables (disease and setting) greatly depend on the organization of the healthcare system. In Spain, the few published studies reveal few changes over the past 8-10 yrs in the reasons for relatives’ satisfaction (symptom control, care continuity, treatment received by the palliative care team) and dissatisfaction (care in emergency departments, lack of equity between rural and urban areas in the access to palliative resources) with the end-of-life care delivered (Cía-Ramos et al., 2012). A good level of satisfaction has been reported, contrasting with accounts of the suffering experienced in accompanying and looking after a dying loved one (Cía-Ramos et al., 2012). According to other studies (Reigada, Ribeiro & Novellas, 2015; Finucane et al., 2020; Ding et al., 2020), experience of care can be influenced by practical issues (admittance, technical assistance, social resources/health) and factors related to relationships (bonds, loss, privacy, support to caregivers, sharing), inner experience (feelings, confrontation strategies, affection, suffering, death, psychological support), and health (symptoms, information on the disease, vulnerability of caregivers).

The research question of the present study was whether the general satisfaction expressed by relatives with end-of-life care agrees with their perception of the lived experience of the care process as a whole. This divide between quantitative measures of patient satisfaction and the lived experience can be explored by adopting a mixed methods approach, bringing together distinct but complementary ways of observing reality (Guba, 1990).

The main objective of this investigation was to explore the perception of caregiver relatives of patients dying from a terminal disease on the care received and the experience lived during the end-of-life care process, based on the results of two studies, one quantitative and the other qualitative, identifying elements of convergence/divergence and developing an integrated interpretation.

Concurrently, a study was conducted on the influence of the type of disease (oncological or non-oncological) and of the care setting (home, emergency department, hospital room, or palliative care unit) on perceptions and lived experiences.

## Materials & Methods

### Design

**Figure 1:**
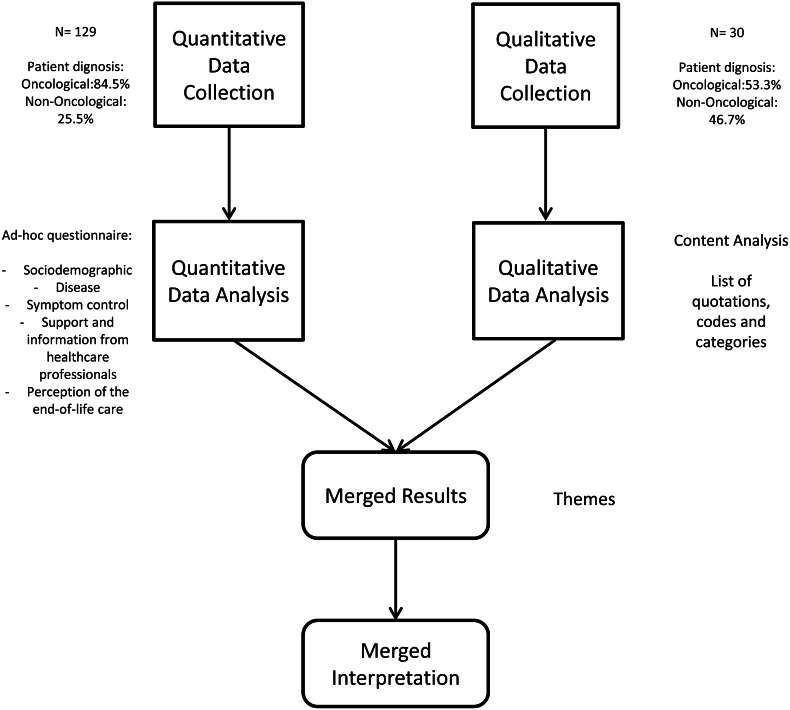
Description of the design.

We conducted an observational, cross-sectional, descriptive, and analytical mixed methods study (Creswell, 2014) (see Fig. 1), following a concurrent triangulation strategy (Creswell & Plano-Clark, 2007) with data integration in the analysis and interpretation (López-Fernández & Molina-Azorín, 2011; Onwuegbuzie & Collins, 2007).

The mixed methods study was based on the quantitative and qualitative results of a previous multicenter study conducted in family caregivers of patients who died from a terminal illness. They completed a questionnaire to assess the perception of the care received and were invited to provide other details of the experience lived through a complementary interview on the main topics addressed in the questionnaire.

A mixed methods approach is invaluable for studying end-of-life care because of the need to consider all aspects, including the overall quality of care perceived (assessable by quantitative instruments) and the subjective experiences of caregiver relatives (better understood with a qualitative design) (Farquhar, Ewing & Booth, 2011). These two approaches may give similar results but can also markedly differ, providing a more comprehensive view of the reality observed (Curry et al., 2013).

### Participants

Participants were selected by intentional sampling, followed by snowball sampling. This method was selected because it can sometimes be difficult to identify an informal caregiver (Barber, 2012) and because relatives are often reluctant to talk about suffering and/or death (Chaturvedi, Loiselle & Chandra, 2009; Pino & Parry, 2019). Participants were initially contacted by the liaison nurse on our team, who briefly explained the nature of the study and invited them to make contact with the research team after obtaining their informed consent (quantitative phase). These participants were invited to a complementary individual interview (qualitative phase) to explore their experience of the end-of-life process. Data collection in the qualitative phase ended when data saturation was achieved.

This study was carried out in Andalusia (Southern Spain), the most populous Spanish Autonomous Community (>8 million inhabitants). The Spanish Healthcare system is a public, universal, and decentralized system in which the Autonomous Communities are responsible for managing and organizing their inhabitants’ health care, including end-of-life care and palliative care‘ (Sociedad Española de Cuidados Paliativos (SECPAL), 2016). According to the latest Atlas of Palliative Care in Europe, Spain is in the third quartile for the number of specialized palliative care services (0.6 per 100,000 inhabitants) and in the first quartile for the consumption of opioids (Arias Casais et al., 2019). Andalusia ranks third among Spanish autonomous regions by the amount of resources for palliative care (Arias Casais et al., 2019).

### Procedure

Selected family members were telephoned by the corresponding unit/department to obtain their verbal consent to participation in the study and to make an appointment for the questionnaire administration. The majority of caregiver relatives who agreed to participate in the study opted to complete the questionnaire at home (participants’ residence), where the interviews were always conducted. After completing the questionnaire, two researchers experienced in qualitative research invited them to add information and comments in their own words. Recording of the interview was refused by the majority of participants to avoid the possibility of their identification. Interview data were collected by one interviewer, using pen and paper, while the other researcher conducted the interview.

### Data collection

The questionnaire was developed with five sections that covered the main aspects of end-of-life care recognized as basic quality indicators according to the reviewed literature, as follows: the sociodemographic characteristics of the relative; the patient and disease; symptom control during past month; support and information from healthcare professionals; and perception of the end-of-life care provided. It contained dichotomous (Yes/No) items and 5-point Likert-type questions that assessed frequency (1= “Never” to 5= “Always”) or degree of satisfaction (1 = “Very Bad” to 5=“Very Good”). This questionnaire has been used in previous research on end-of-life care (Guardia-Mancilla et al., 2017; Guardia Mancilla et al., 2018). It took between 18 and 25 min to complete the questionnaire.

The complementary interview included eight questions on the experience of the relative at different stages of the end-of-life process, including the emotional response to the diagnosis, prognosis, and death, the information received, and their satisfaction with the healthcare delivered. Interviews were conducted in a single 15–30 min session.

### Data analysis

After a descriptive analysis of questionnaire results (means, standard deviations, frequencies), Pearson’s Chi-square or the Student *t*-test for independent samples was applied, as appropriate. SPSS version 19.0 was used for the data analyses, and *p* values <.05 were considered significant.

Transcriptions and notes of the interviews were analyzed by two researchers, who prepared the initial and emerging codes by consensus. Following the recommendations for use inductive content analysis of Elo & Kyngäs (2008) these citations were assigned codes by open encoding and creating the main categories.

A content analysis was then conducted by researchers using Altas.ti version 7.2 and was subjected to a triangulation process in which various interviews were independently coded by two different researchers.

Quantitative and qualitative data were integrated using a concurrent triangulation design (Creswell et al., 2003), following a strategy of comparison around the main topic categories addressed in the questionnaires and interviews (Combs & Onwuegbuzie, 2010). Four themes emerged from the integration of quantitative data and qualitative categories. Cross-sectional codes for the type of disease and place of death were considered in the comparative analysis. The components of the triangulation are specified in Table 1.

**Table 1:** Components of concurrent triangulation.

**Themes Concurrent** **Triangulation**	**Questionnaire** **Items**	**Interview** **Categories**	**Cross-sectional Codes** **comparative analysis**	
1. Communication and Information	Situation and prognosis	Diagnostic communication	Patient disease	Oncological Non-oncological
	Available resources	Prognosis information		
	Different therapeutic options	Communication with professionals	Place of death	Hospital room ICU/Emergency Palliative care Home
	Comfort measures			
2. Professional support	Perceived professional support	Attention received in the last month		
	Nursing support	Nursing support at home		
	Physician support	Palliative care support at home		
	Commenting concerns with professionals			
3. Symptom control	General symptom control	Disease process: suffering		
	Pain control	Delay in attention		
	Best controlled symptoms	Professional Care at Home		
	Worst controlled symptoms			
			
4. Perceived experience of death and end of life process	Quality of experience	Perceived quality and experience of the process of dying		
			
	Unmet needs	Awareness about the proximity of death		
	Aspects of improvement identified	Bereavement Process		
		Conditions during the last days of life		

### Ethical considerations

The research project of which this study forms a part was approved and authorized by the Ethics and Clinical Research Committees of the participating hospitals: Virgen de las Nieves Hospital, San Cecilio Clinical Hospital and Baza Hospital. All participants signed their informed consent to participate in the study, which followed the principles of the Helsinki Declaration (2013) and complied with Spanish law (Organic Law 15/1999) on personal data protection, guaranteeing the anonymity of relatives and patients. They were told that they could withdraw from the study at any point. Questionnaires and interviews were identified with an alphanumeric code, removing all references to individuals or places from interview transcripts.

## Results

The eligible study population comprised family members of patients dying from a terminal disease between January 1st and November 30th 2016 and attended in five public healthcare centers located in Southern Spain. The inclusion criteria were: age >18 yrs, family relationship with a patient dying from advanced and/or terminal disease within the past 6 months, direct involvement in the end-of-life care of the patient, and signed consent to voluntary participation in the study. The exclusion criteria were: difficulty in remembering information in the interview or being in a state of severe despondency.

A total of 129 relatives were enrolled in the study and completed the questionnaire (quantitative phase) (Table 2). Their age ranged from 19 to 82 years, with a mean of 47.81 years (SD = 15.31); 83.7% were female and 59.7% were offspring of the deceased. With respect to the patients, 84.5% died from an oncological disease. Although half (49.9%) of the patients were at home for their last month of life, only 24.8% died there, with 41.9% dying in a hospital bed, 24.8% in a palliative care unit, and 8.5% in the ICU. Thirty relatives agreed to participate in the interview (qualitative phase) (see Table 2): 53.3% (*n* = 14) were relatives of patients with non-oncological disease and 46.7% (*n* = 16) were relatives of patients with oncological disease.

Findings of the quantitative and qualitative phases were integrated, considering the quantitative/qualitative methodology applied, the oncological/non-oncological disease of the patient, and the place of death. The questionnaire results show that the end-of-life period was shorter for oncological versus non-oncological patients. The place of death did not differ as a function of disease type, but the last month of life was more frequently spent at home by those with oncological versus non-oncological disease, with fewer hospital admissions during this period (Table 3).

### Theme 1: communication and information

According to the results of the quantitative phase, most participants (79.7%) were always or almost always informed on the status and prognosis of their relative and received information on available resources (74.4%), therapeutic options (79.8%), and comfort measures (69.8%) Information on available therapeutic options appears to have been better for relatives of patients with oncological versus non-oncological disease, but no difference between these groups was observed in information about the diagnosis and prognosis or on available comfort measures and resources (see Table 3).

**Table 2:** Sociodemographic characteristics of participants.

**Participant characteristics**	**Questionnaire** (*n* = 129)	**Interview** (*n* = 30)	**Statistics**
	**N or Mean (% or SD)**	**N or Mean (% or SD)**	t or *χ*^2^
Mean age (yrs)	47.81 (15.31)	53.30 (13.74)	*t*(157) = 1.80, *p* = .074
Sex			*χ*^2^(1) = .16, *p* = .690
Male	21 (16.3%)	4 (13.3%)	
Female	108 (83.7%)	26 (86.7%)	
Kinship			*χ*^2^(5) = 3.64, *p* = .602
Offspring	77 (59.7%)	14 (46.7%)	
Spouse	32 (24.8%)	11 (36.7%)	
Grandchild	5 (3.9%)	1 (3.3%)	
Sibling	4 (3.1%)	0	
Father/Mother	2 (1.6%)	1 (3.3%)	
Other^*^	9 (7%)	3 (10%)	
**Patient characteristics**			
Patient disease			*χ*^2^(1) = 14.06, *p* < .001
Oncological	109 (84.5%)	16 (53.3%)	
Non-oncological	20 (15.5%)	14 (46.7%)	
Time course in months			
Oncological	20.10 (23.97)	32.81 (29.59)	*t* (123) = 1.92, *p* = .057
Non-oncological	45.85 (29.37)	60.43 (21.63)	*t* (32) = 1.58, *p* = .124
Place of longest stay (in last month)			*χ*^2^(1) = 17.37, *p* < .001
Home	63 (49.1%)	3 (10%)	
Hospital	67 (51.9%)	27 (90%)	
Place of death			*χ*^2^(3) = 14.87, *p* = .002
Hospital room	54 (41.91%)	13 (43.3%)	
Palliative care	32 (24.8%)	16 (53.3%)	
Home	32 (24.8%)	0
ICU/Emergency	11 (8.5%)	1 (3.3%)	

**Notes.**

* Includes relatives of the third degree or more by consanguinity.

**Table 3:** Characteristics of the attention received according to the type of disease (oncological or non-oncological) in the questionnaire.

**Variables**	**Oncological. (*n* = 109)**	**Non-Oncological (*n* = 20)**	**Statistics**
	**Mean or N (SD or %)**	**Mean or N (SD or %)**	
Sex			*χ*^2^ (1) = 3.27 *p* = .071
Male	15 (13.80%)	6 (30%)	
Female	94 (86.20%)	14 (70%)	
Mean age in yrs (SD)	46.88 (15.43)	52.90 (13.89)	*t* (127) = 1.63 *p* = .104
Kinship			*χ*^2^ (5) = 1.57 *p* = .905
Offspring	65 (59.60%)	12 (60%)	
Spouse	26 (23.90%)	6 (30%)	
Grandchildren	4 (3.70%)	1 (5%)	
Siblings	4 (3.70%)	0	
Father/Mother	2 (1.80%)	0	
Other	8 (7.30%)	1 (5%)	
Time course of the disease in months	20.10 (23.97)	45.85 (29.37)	*t* (127) = 4.26 *p* < .001
Place of death			*χ*^2^ (2) = 5.89 *p* = .053
Hospital	75 (68.80%)	19 (95%)	
Home	30 (27.5%)	1 (5%)	
Other	4 (3.70%)	0	
Spent the last month			*χ*^2^ (1) = 12.94 *p* < .001
At home	64 (58.7%)	3 (15%)	
In hospital	45 (41.3%)	17 (85%)	
Died where they wanted			*χ*^2^ (2) = 7.26 *p* = .027
Yes	32 (29.40%)	3 (15%)	
No	36 (33%)	3 (15%)	
Don’t know	41 (37.60%)	14 (70%)	
Pain was controlled			*χ*^2^ (2) = 2.96 *p* = .227
Almost always	76 (59.80%)	14 (11%)	
Sometimes	19 (15%)	5 (3.90%)	
Very few times	13 (10.20%)	0	
Visits to the emergency department	1.94 (5.80)	3.15 (5.45)	*t* (127) = .86. *p* = .390
Hospitalizations during the last month	.94 (.863)	1.5 (1.28)	*t* (126) = 2.43 *p* = .016
Domiciliary Primary Care visits	2.55 (5.28)	3.40 (5.95)	*t* (126) = .65 *p* = .516
Domiciliary Palliative Care visits	1.20 (2.38)	0 (0)	*t*(107) = − 5.26*p* = .001
Visits to the Palliative Care unit	.67 (2.18)	0 (0)	*t*(108) = − 3.21*p* = .002
Information on the status and prognosis of their relative			*χ*^2^ (2) = .801 *p* = .670
Almost always	88 (80.70%)	14 (73.70%)	
Sometimes	11 (10.10%)	2 (10.50)	
Very few times	10 (9.20)	3 (15.80%)	
Information on the therapeutic options			*χ*^2^(1) = 5.79*p* = .016
Yes	91 (83.50%)	12 (60%)	
No	18 (16.50%)	8 (40%)	
Information on comfort measures			*χ*^2^(1) = .01*p* = .980
Yes	76 (69.7%)	14 (70%)	
No	33 (30.30%)	6 (30%)	
Information on resources available for patient care			*χ*^2^(1) = 2.43*p* = .622
Yes	82 (75.20%)	14 (70%)	
No	27 (24.80%)	6 (30%)	

In the qualitative phase, participants described the time they were informed of the diagnosis as traumatic and/or negative, and they described the language used as ambiguous and the information given by professionals as scant. Relatives of oncological patients were better informed and had greater knowledge of the disease severity. Relatives of non-oncological patients reported their distress on receiving the information, which was usually given just before or even after the death (see Table 4).

**Table 4:** Quotations regarding communication, symptom control, professional support and perceived experience of death.

**Main Theme**	**Quotes Oncological Patients**	**Quotes Non-Oncological Patients**
1. Communication and Information	*”We lacked quite a lot of information about everything that was happening, of course...what was happening to my husband was no mystery, it had a name and it was cancer...and people with cancer die...it is another stage, it doesn’t have to be prettified or referred to as “his situation”...”* (Oncological, C02).	*…”I remember two doctors came out and approached us to give us information about what was happening …The doctors looked at us and said that my uncle had worsened and that he had to be better controlled …that they were going to transfer him to the ICU and that he would be better controlled there …I thought…well if he is better in the ICU, well, better …I never thought he was going to die there …”* (Non-oncological, C20).
2. Professional support	*…”My husband spent almost the whole of the disease at home, and in the end it was a doctor and a nurse who came home to see him …” (Oncological, C03).* *…”My father was diagnosed with prostate cancer for four years and died two months ago. He was at home, attended by the palliative care team, until he had to be hospitalized …” (Oncological, C07)*	*…”We had oxygen at home and a nurse came every week to see how he was. To be honest, the nurse of the healthcare center was very good because she came to see him when she could for his controls …” (Non-oncological, C05).*
3. Symptom Control	*”Day after day going to the emergency department...and they didn’t tell him anything or give him any solution for what he had. My father has always been very strong, he has never complained about anything, but it was complicated because he complained a lot, his belly was very swollen, and he urinated blood... We were waiting for him to get surgery for some cysts he had in his bladder and that’s why they didn’t tell him or do anything...he had to wait for the day of the surgery. They only prescribed morphine to ease the pain a bit …“* (Oncological, C01).	*”First he began not being able to breathe properly and it got worse with time. He had a lung disease that worsened over time. The doctor prescribed oxygen to have at home, because he was often suffocated …”* (Non-oncological, C18). *”My grandmother always complained about stomachache, a lot of pain, and her belly was always swollen. It was almost 6 years of pain in her abdomen...”* (Non-oncological, C16).

### Theme 2: professional support

In the quantitative phase, the relatives generally reported feeling always or almost always supported by healthcare professionals (especially nurses), and 77.4% of them were always/almost always able to talk about their concerns with professionals.

Professional support was more highly rated by relatives of patients who died in a palliative care unit or at home (Table 5). Likewise, a high percentage of relatives felt able to comment on their concerns with professionals at home, in a palliative care unit, or in a hospital room, whereas this appeared to be rarely or never possible in an emergency department or ICU. Relatives also reported receiving more information at home (see Table 5).

**Table 5:** Perception of professional support and information as a function of patient location.

Professional support		Home	Emergency department/ ICU	Hospital room	Palliative care	Statistics
			*n*(%)	*n*(%)	*n*(%)	*n*(%)
Perceived professional support *n* = 128	Always/Almost always	26 (81.3%)	5 (45.5%)	41 (77.4%)	30 (93.8%)	*χ*^2^(1) = 18.45 *p* = .005
	Sometimes	5 (15.6%)	2 (18.2%)	4 (7.5%)	2 (6.3%)	
	Very few times/Never	1 (3.1%)	4 (36.4%)	8 (15.1%)	0 (0%)	
Nursing support *n* = 129	Yes	10 (31.3%)	2 (18.2%)	24 (44.4%)	17 (53.1%)	*χ*^2^(6) = 7.42 *p* = .284
	No	1 (3.1%)	0 (0%)	3 (5.6%)	1 (3.1%)	
	Not specified	21 (65.6%)	9 (81.8%)	27 (50%)	14 (43.8%)	
Physician support *n* = 129	Yes	6 (18.8%)	1 (9.1%)	13 (24.1%)	17 (53.1%)	*χ*^2^(6) = 14.08 *p* = .029
	No	8 (25%)	3 (27.3%)	13 (24.1%)	3 (9.4%)	
	Not specified	18 (56.3%)	7 (63.6%)	28 (51.9%)	12 (37.5%)	
Discussing concerns with professionals *n* = 128	Always/Almost always Sometimes Very few times/Never	30 (93.8%)	4 (36.4%)	39 (73.6%)	26 (81.3%)	*χ*^2^(6) = 29.72 *p* = < .001
		2 (6.3%)	1 (9.1%)	7 (13.2%)	5 (15.6%)	
		0 (0%)	6 (54.5%)	7 (13.2%)	1 (3.1%)	
Professional information						
On situation and prognosis *n* = 128	Always/Almost always	30 (93.8%)	6 (54.5%)	42 (79.2%)	24 (75%)	*χ*^2^(6) = 12.40 *p* = .054
	Sometimes	1 (3.1%)	3 (27.3%)	7 (13.2%)	2 (6.3%)	
	Very few times/Never	1 (3.1%)	2 (18.2%)	4 (7.5%)	6 (18.8%)	
On available resources *n* = 129	Yes No	29 (90.6%)	4 (36.4%)	38 (70.4%)	25 (78.1%)	*χ*^2^(3) = 13.48 *p* = .004
		3 (9.4%)	7 (63.6%)	16 (29.6%)	7 (21.9%)	
On the different therapeutic options *n* = 129	Yes No	31 (96.9%)	6 (54.5%)	41 (75.9%)	25 (78.1%)	*χ*^2^(3) = 10.72 *p* = .013
		1 (3.1%)	5 (45.5%)	13 (24.1%)	7 (21.9%)	
On comfort measures *n* = 129	Yes	25 (78.1%)	7 (63.6%)	34 (63%)	24 (75%)	*χ*^2^(3) = 2.86 *p* = .414
	No	7 (21.9%)	4 (36.4%)	20 (37%)	8 (25%)	

In the qualitative interviews, most relatives of oncological patients described being always or almost always supported by professionals, while most relatives of non-oncological patients described only sometimes receiving this support (see Table 4).

In relation to the end-of-life setting, greater professional support was always or almost always felt by relatives in palliative care units or at home, somewhat less frequently in hospital rooms, and much less frequently in emergency departments or ICUs. In the quantitative phase, a high percentage of participants were always or almost always able to talk about their concerns with professionals at home (93.8%) or in a palliative care unit (81.3%) or hospital room (73.6%), but this was described as rarely or never possible in an emergency department or ICU (see Table 5).

In this phase, domiciliary care was perceived as scant, except in the case of oncological patients during the end-of-life period. However, high satisfaction was expressed by relatives of non-oncological patients who received care at home when their functions became more limited (Table 4).

### Theme 3: symptom control

According to results obtained in the quantitative phase, the most frequently reported symptoms of patients were pain (89.9%) and weakness (74.8%), followed by dyspnea (45.3%) and fever (42.3%). When present, pain (66.4%) and fever (53.8%) were the most successfully controlled symptoms.

Symptom control varied as a function of the place in which the last month of life was spent, being best when at home or in a palliative care unit and worst when in emergency department or ICU. At home, the best controlled symptoms were pain and dyspnea and the worst controlled were confusion and weakness/tiredness. Fever was best controlled in hospital rooms, while more complex symptoms such as weakness/tiredness and confusion were best controlled in palliative care units. Symptoms were worst controlled in ICUs and emergency departments (see Table 6).

**Table 6:** Symptom control in terms of the location of the patients.

Symptom control		Home	Emergency department/ICU	Hospital room	Palliative care unit	Statistics
		*n*(%)	*n*(%)	*n*(%)	*n*(%)	
General symptom control *n* = 129	Always/Almost always	26 (81.3%)	3 (27.3%)	31 (57.4%)	26 (81.3%)	*χ*^2^(6) = 29.72 *p* = .008
	Sometimes	4 (12.5%)	5 (45.5%)	17 (31.5%)	4 (12.5%)	
	Very few times/Never	2 (6.3%)	3 (27.3%)	6 (11.1%)	2 (6.3%)	
Pain control *n* = 127	Always/Almost always	28 (87.5%)	5 (50%)	31 (58.5%)	26 (81.3%)	*χ*^2^(6) = 12.85 *p* = .045
	Sometimes	2 (6.3%)	3 (30%)	14 (26.4%)	5 (15.6%)	
	Very few times/Never	2 (6.3%)	2 (20%)	8 (15.1%)	1 (3.1%)	
Best controlled symptoms	Dyspnea	8 (53.3%)	3 (33.3%)	10 (23.8%)	7 (21.9%)	*χ*^2^(6) = 21.76 *p* = .001
	Weakness/Tiredness	3 (9.4%)	1 (11.1%)	6 (11.8%)	10 (31.3%)	*χ*^2^(6) = 22.15 *p* ≤ .001
	Vomits	0 (0%)	0 (0%)	9 (17.6%)	8 (25%)	*χ*^2^(6) = 27.22 *p* = < .001
	Pain	29 (90.6%)	6 (54.5%)	32 (59.3%)	17 (53.1%)	*χ*^2^(6) = 14.10 *p* = .029
	Confusion	3 (9.4%)	0 (0%)	2 (3.8%)	4 (12.5%)	*χ*^2^(6) = 5.91 *p* = .434
	Fever	1 (3.1%)	1 (9.1%)	20 (37.0%)	6 (21.40%)	*χ*^2^(6) = 30.56 *p* ≤ .001
Worst controlled symptoms	Dyspnea	1 (3.1%)	4 (44.4%)	12 (24%)	4 (12.5%)	*χ*^2^(6) = 17.62 *p* = .007
	Weakness/Tiredness	8 (25.0%)	6 (54.5%)	23 (42.6%)	11 (34.4%)	*χ*^2^(6) = 21.59 *p* ≤ .001
	Vomits	2 (6.3%)	0 (0%)	4 (7.8%)	6 (19.4%)	*χ*^2^(6) = 25.86 *p* ≤ .001
	Pain	2 (6.3%)	4 (36.4%)	18 (34%)	13 (40.6%)	*χ*^2^(6) = 20.82 *p* = .002
	Confusion	3 (37.5%)	4 (100%)	11 (52.4%)	9 (69.2%)	*χ*^2^(3) = 5.53 *p* = .137
	Fever	1 (3.1%)	0 (0%)	4 (8%)	6 (18.8%)	*χ*^2^(6) = 24.96 *p* = < .001

The qualitative phase revealed that not all oncological patients were in a specific area for symptom control during the last month of life, and highlighted the suffering caused to relatives by their perception of severe symptoms. Symptom control was perceived in a different manner by relatives of patients with non-oncological diseases, which they described as developing over years, with a progressive worsening of symptoms (see Table 4).

### Theme 4: Perceived experience of death and end-of-life process

The perceived experience of death was related to the needs that were attended. Thus, in the quantitative phase, 83.3% of participants who reported a good or very good experience of attention to their patient’s needs felt that all needs were attended to, while half of the relatives did not describe any aspect that required improvement. Needs had been met even when the experience had been poor (45.7%), although those reporting a poor experience felt that attention to needs and professional support should be improved. In fact, this was the aspect most frequently described as requiring improvement: by 52.4% of those reporting a poor or very poor experience and 42.9% of those reporting a good or very good experience. Other aspects needing improvement were bereavement support and conditions to promote end-of-life care.

Regarding the attention to needs and professional support, the interviews revealed the need for improvements in the information provided and, in the language used, which was sometimes considered ambiguous language. Unattended needs were mainly related, by both oncological and non-oncological patients, to the final stage of life. Differences were found between the relatives of oncological and non-oncological patients, with the former having an individual room, offering privacy during the last days of their life and allowing relatives to be with them to the very end. This was not the case for the non-oncological patients (Table 7).

**Table 7:** Quotations of Sub-codes in Theme 4 Perceived experience of death and end of life process.

Subcode	*Oncological*	*Non-Oncological*
Perceived Quality and experience of the process of dying	*“The information they gave us was always scarce and as well as being very limited we couldn’t understand it... Why so much mystery? Didn’t they know what was happening with my father or had they forgotten to tell us?”* (Oncological, C01). *…”The doctors were very clear with us, they told us that she had a cancer that was not compatible with life...At first you are in shock, but when you see that she improves little by little, you think “maybe it isn’t as bad as they say”... (Oncological, C28).*	*”No need was met, except at the last moment when I begged “please don’t let her suffer any more” and she was sedated …”* (Non- oncological, C16). *“There was always a lack of information; they informed us, of course they informed us, but I listened to the doctor and I didn’t understand anything he said…”* (Non- oncological, C20). *…”We did not stay at night during the stay, but from that Monday we did... we told our mother that it was not necessary for her to stay but she’s still sorry for not having done so to be able to bid farewell to my father...” (Non-oncological, C14).*
Awareness about the proximity of death	*…” for me it was living with the uncertainty of not knowing when she would die and I could not make the most of my time with her…”* (Oncological, C27)	*“She was in a shared room …I remember the relatives of the other woman, who didn’t stop looking at my mother, because of her weight…you know…it made me angry and I asked them if they wanted something …[...] They sedated her and my mother died three days later…She was surrounded by all of us but we had no privacy…”* (Non-oncological, C10).
Bereavement Process	*…”The day he died we were all in the room, around the bed, we wanted to stop in time every last breath he took to be able to have him more time by our side...but that air stopped going in and my husband stopped breathing...we had lost him...”* (Oncological, C03) *“The whole family were gathered in a room when they told us that my uncle had little time left and that they would do anything possible for him not to suffer…It was a painful but nice farewell, because we had the opportunity to do so …”* (Oncological, C22).	*…”I hope she can forgive me, because every day of my life I regret it, letting her die in hospital …” (Non-oncological, C16).* *…”We didn’t have the opportunity to say goodbye to him, give him a hug…nothing…they didn’t let us say farewell to him…and all because of the damned timetables…The timetables meant that we were not with my uncle, accompanying him so that he wasn’t alone…”* (Non-oncological, C20).
Conditions during the last days of life	*“Some days before he died, the doctor said that they had to give him stronger medication to have an effect but that this would leave him asleep. I accepted it because my husband was suffering quite a lot...it was unbearable. Is it necessary to have to suffer so much to die?* (Oncological, C30). *“We were in the oncology department for a long time, we knew everyone, and when he was going to die, they moved him to another department”* (Oncological, C11).	*My wife went to palliative care unit and spent two days there .. She died sedated and surrounded by us all. If this service is available, it is incredible that they wait to the last moment to use it.” (Non-oncological, C15).* *…”It was very sad to see her like that after everything...surrounded by a lot of people in a room and “murmuring” about her...My mother deserved to die in an individual room and being able to say goodbye to her as she deserved...” (Non-oncological, C25).*

In the quantitative phase, the relatives‘ perception of their experience of death was also related to the place in which it concurred, with the experience considered good or very good when at home by 84.4% or in a palliative care unit by 71.9% and considered poor or very poor in the emergency department/ICU (75%).

The qualitative phase showed that the differentiation by relatives between a good or bad death was not only related to their perception of suffering, pain and symptom control but also to the attitude of the staff and the adequacy of the place of death for accompanying the patient to the end.

With regard to pain and suffering, patients died under sedation to minimize their suffering. In a minority of cases sedation was not initiated until requested by family members. The experience by relatives of the suffering endured by a dying loved one was described as changing their attitude to the quality of death. They considered the idea of dying slowly and painfully as unbearable.

Concerns of relatives about care during the bereavement process were related to the difficulty in expressing their emotions, diagnostic concerns, lack of symptom control, and the fact that the patient did not die in the preferred place, among others (Table 7). Participants expressed concern and regret for not having more time to bid their loved one farewell and for having failed to respect his wish to die at home.

**Figure 2:**
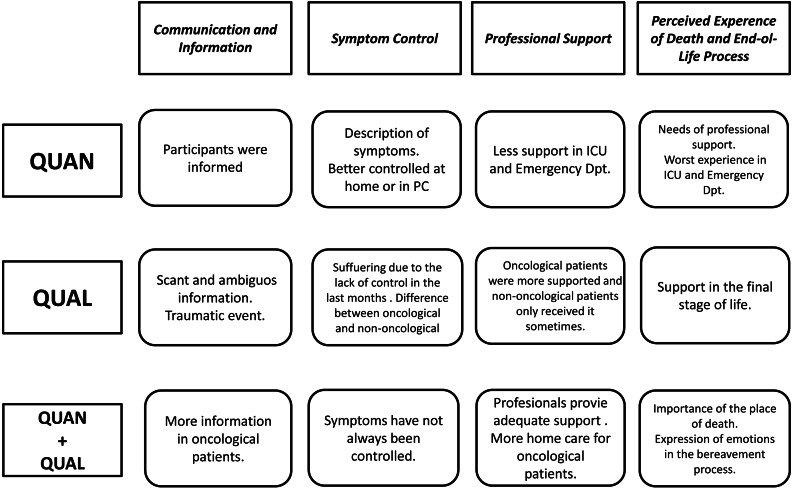
Integration of main qualitative and quantitative results.

Finally, an integration of the main results in the four main themes is depicted in Fig. 2.

## Discussion

This mixed methods study analyzed perceptions of the care received by relatives of patients at the end of their life as a function of the oncological or non-oncological nature of the disease, the setting for the last month of life, and the place of death. The quantitative and qualitative phases provided complementary information and also revealed some discrepancies between perceptions and experiences expressed in the interviews and questionnaires.

A main study finding was the difference between relatives of oncological and non-oncological patients in their communication with healthcare professionals, with the former being better informed on the severity of the patients’ situation. Previous studies have indicated that palliative care appears to be delivered at the end of life in a more consistent and unambiguous manner in the case of oncological patients (Hewison, Lord & Bailey, 2015; Leong, 2015). It is possible that professionals can diagnose the end-stage of disease with greater confidence in oncological patients, allowing them to inform relatives about the severity of the patient in a clearer way. Relatives may also be more aware of the possibility of death from cancer that from non-oncological disease and may therefore be more prepared to receive this information (Arrighi, Jovell & Navarro, 2010).

It can be highly challenging to predict the progression of patients with advanced chronic disease (Gill et al., 2010), making it difficult for professionals to know how to describe the severity of the situation. Relatives who have previously observed major relapses or severe episodes of the disease in the patients may tend to minimize the gravity of the situation and maintain their hopes for a recovery. Hence, even when appropriate information is well provided, the perception of relatives may be more influenced by their previous experience of the disease (Aldasoro et al., 2012; Gallagher & Krawczyk, 2013).

Relatives of oncological and non-oncological patients also differed in their perception of the support received in distinct settings. Thus, the support of healthcare professionals was considered greater when the patient was in hospital by relatives of oncological patients but when the patient was at home by relatives of non-oncological patients. No such differences were observed in relation to palliative care units, in line with previous studies (Gallagher & Krawczyk, 2013).

Aldasoro et al. (2012) reported that relatives of non-oncological patients felt more supported at home, where they can participate in the care of their relatives. When the disease becomes more severe and patients are hospitalized, healthcare professionals take on their care responsibilities, leaving relatives with a secondary care role (Virdun et al., 2015). The relatives of oncological and non-oncological patients also differed in the attention to needs and comfort measures depending on whether the patient was in hospital or at home. Oncological patients more frequently spent the last month of life at home, with a reduction in the number of hospital admissions during this period, which might be facilitated by visits from palliative care support teams (Vega et al., 2011).

Conditions for end-of life care tend to be superior in palliative care units than in other hospital departments, which generally offer less privacy to patients, who can be in small rooms with multiple occupants (Hewison, Lord & Bailey, 2015; The Economist Intelligence Unit, 2015) and are less prepared to address this situation (Virdun et al., 2015). Among hospital departments, professionals in oncology departments are considered more sensitive to the needs of patients and their relatives during the end-of-life process (Bonilla, Segovia & Saltos, 2015).

The experience of death perceived by the relatives was described as less traumatic when the patients’ pain was controlled, when they received adequate information, and when they could accompany them in their final moments in a satisfactory manner. Krikorian et al. (2010) reported that the experience of relatives at the time of death was more positive when they received support from the attending healthcare professionals. Other recent studies emphasized that the experience of relatives is more positive when the death occurs in the place preferred by the patient (Hoare et al., 2015; Raijmakers et al., 2018).

Finally, the experience of accompanying patients during the end-of-life process seemed to be determined by the expectations of relatives. Thus, the worst experiences were described by relatives who had not been informed that the patient’s condition was so severe and/or who experienced inadequate support for their bereavement. Various authors have emphasized the need for improvements in the psychological support offered to caregiver relatives during and after the death of patients (Jacobs et al., 2017; Schumacher et al., 2008).

As recommendations for clinical practice, the perception of care and the experience lived by the relatives in this study highlight the need for a broader approach to end-of-life care in general and palliative care in particular, as called for by the WHO (World Health Organization, 2014; Gómez-Batiste et al., 2017). From this point of view both, end-of-life and palliative care should be applied early in chronic pathologies, without distinghuish whether or not the disease is oncological or non-oncological (World Health Organization, 2014; Gómez-Batiste et al., 2017). Likewise, the training of professionals who work with these patients needs to be strengthened and the clinical criteria for end-of-life care need to be adapted, so that patients and relatives suffer no discrimination due to the nature of the disease or the place where they are treated. Professionals working with patients at the end of life should address deficiencies in the current provision of palliative care and support an increased recognition of the place, role and experience of family caregivers.

### Limitations of the study

One potential limitation of this study was the *ad hoc* design of the questionnaire, although it was based on a detailed review of the literature (Barbera, Paszat & Chartier, 2006; Earle et al., 2005; Van den Block et al., 2008). Further research is required to verify the usefulness and psychometric properties of this instrument for evaluating the quality of end-of-life care. In addition, it was not possible to study homogeneous samples of oncological and non-oncological patients, and participants in the qualitative and quantitative phase differed in some of the sociodemographic characteristics. This is largely attributable to the difficulty of identifying non-oncological patients in end-of-life processes in our healthcare system (Campos-Calderón et al., 2017). Finally, because most relatives refused to be recorded, analysis of the interviews was based on written notes taken by researchers, and the appropriate caution should be taken in interpreting the results.

### Future outcomes

The present research has a number of future outcomes. First, it identifies highly relevant situations for delivering quality care to patients and their families in an end-of-life situation, which have already been reported to the health authorities. Second, it points out differences in end-of-life processes according to the type of disease and place of treatment, highlighting the need for specific measures and even the modification of protocols for health centers and home care. Finally, it promotes dissemination of the experiences and perceptions of caregiver relatives as one element of the triangle sustaining health care and as a means to exteriorize clinical relationships and care management.

## Conclusions

In conclusion, the experience of the relatives of patients in end-of life care differed as a function of the oncological or non-oncological disease of the patient and the place in which they spent the last month of their life. Relatives of oncological patients described a more positive experience in relation to communication with healthcare professionals and the information and support received when the patient was attended in hospital, especially in a palliative care unit; in contrast, relatives of non-oncological patients reported a more positive experience of support from healthcare professionals when the patients was attended at home.

Combined analysis of the results obtained in the quantitative and qualitative phases reinforced and complemented the results obtained with each method. In general, the response of relatives were more positive when selecting responses in the questionnaire than when responding to open questions in the interview, when they tended to be more critical and negative.

##  Supplemental Information

10.7717/peerj.10516/supp-1 Supplemental Information 1 Raw data

10.7717/peerj.10516/supp-2 Supplemental Information 2 Questionnaire (English)

10.7717/peerj.10516/supp-3 Supplemental Information 3 Original Questionnaire (Spanish)

## Additional Information and Declarations

### Data Availability

The following information was supplied regarding data availability: Data is available in the Supplementary Files.
