# Supplementary material for: Experiences of family caregivers of patients with terminal disease and the quality of end-of-life care received: a mixed methods study
Source: PeerJ. 2020 Dec 14;8:e10516. doi: 10.7717/peerj.10516 (PMC7745673; doi:10.7717/peerj.10516)
Supplement: Supplemental Information 2 [file peerj-08-10516-s002.doc]

FAMILY QUESTIONNAIRE

CODE:

| _ _ _ | _ _ | _ _ _ |
| --- | --- | --- |
| HOSPITAL[[1]](#footnote-2) | ID[[2]](#footnote-3) | CASE Nº |

1. **Respondent’s socio-demographic characteristics**
   1. Age……………
   2. Gender: 🗌 Male 🗌 Female
   3. Location…………………………
   4. Marital status: 🗌 Single 🗌 Married 🗌 Divorced 🗌 Widowed
   5. Relationship with the deceased

🗌 Partner 🗌 Son/Daugther 🗌 Father/mother 🗌 Brother/Sister 🗌Grandson/Granddaugther 🗌 Other (specify)……………………….…

1. **Pacient’s socio-demographic characteristics**
   1. Age……………
   2. Gender: 🗌 Male 🗌 Female
   3. Location…………………………
   4. Marital status: 🗌 Single 🗌 Married 🗌 Divorced 🗌 Widowed
   5. Disease he/she was suffering………………………………………………………………………………
   6. Evolution of the disease over time:

Days ......

Months ……

Years ……

- 1. Time elapsed since the Decedent's death:

Days ......

Months ……

- 1. Place of death:

🗌 Family residence 🗌 Hospital 🗌 Bed Room 🗌 ICU

🗌 Emergency room 🗌 Palliative Care 🗌 Other (specify)…………………………..

1. **Data on the last month**
   1. During the last month your relative spent nearly all his/her time: 🗌 At home 🗌 Hospitalized 🗌 Nursing Home

**AT HOME / NURSING HOMER:**

- 1. Number of emergency visits:…………

Duration ………. Cause……………………………………………………………………..…

Duration ………. Cause……………………………………………………………………..…

Duration ………. Cause……………………………………………………………………..…

Duration ………. Cause……………………………………………………………………..…

- 1. Number of hospital admissions:…………

Duration ………. Cause……………………………………………………………………..…

Duration ………. Cause……………………………………………………………………..…

Duration ………. Cause……………………………………………………………………..…

- 1. Home care by the Primary Care Team

Number of follow-up and monitoring visits: ……….

Number of occasional visits under request: ……….

- 1. Home Care by the Palliative Care Team

Number of follow-up and monitoring visits: ……….

Number of occasional visits under request: ……….

- 1. Number of telephone consultation with the Palliative Care Team:……….

[Reason for consultation](https://www.linguee.es/ingles-espanol/traduccion/reason+for+consultation.html)……………………………………………………

………………………………………………………………………………………………………..

- 1. Number of visits to the Palliative Care Unit:……….

[Reason for the](https://www.linguee.es/ingles-espanol/traduccion/reason+for+consultation.html) visit……………………………….……………………

**HOSPITALIZED:**

- 1. Number of visits of Palliative Care Professionals:……..…

Cause ……………………………………………………………………..……..………………

Cause ……………………………………………………………………..……..………………

Cause ……………………………………………………………………..……..………………

Cause ……………………………………………………………………..……..………………

Cause ……………………………………………………………………..……..………………

- 1. Number of transfers to ICU:…………

Cause ……………………………………………………………………..……..………………

Cause ……………………………………………………………………..……..………………

Cause ……………………………………………………………………..……..………………

- 1. Tests Performed
- Biopsy
- Xrays
- Ultrasound scan
- CT scanning
- MRI scan (magnetic resonance)
- Endoscopy
- Electrocardiogram
- Blood tests
- Urine tests
- Puncture
- Other: ___________________
  1. Therapeutic measures taken

🗌 Chemotherapy/ radiotherapy 🗌 Dialysis 🗌 Nasogastric tube 🗌 Parenteral nutrition

🗌 Urinary catheter 🗌 Saline 🗌 Mechanical ventilation 🗌 Other (specify)………………………

1. **During the last month**
   1. Symptoms Manifested:

🗌 Dyspnea 🗌 Vomits 🗌 Confusion 🗌 Weakness/ Fatigue 🗌 Pain

🗌 Fever 🗌 Others (specify)…………..…………

- 1. In general, ¿ **Do you consider that the symptoms that appeared were controlled?**?

🗌 Always 🗌 Most of the time 🗌 Sometimes

🗌 Rarely 🗌 Never 🗌 Don’t know/Don’t answer

- 1. In general, do you think the pain was controlled?

🗌 Always 🗌 Most of the time 🗌 Sometimes

🗌 Rarely 🗌 Never 🗌 Don’t know/Don’t answer

- 1. Of the symptoms that your family member manifested, which symptoms were **better** controlled?

🗌 Dyspnea 🗌 Vomits 🗌 Confusion 🗌 Weakness/ Fatigue

🗌 Pain 🗌 Fever 🗌 Asthenia (chronic fatigue) 🗌 Others (specify)…………..…………

- 1. Of the symptoms that your family member manifested, which symptoms were **worse** controlled?

🗌 Dyspnea 🗌 Vomits 🗌 Confusion 🗌 Weakness/ Fatigue

🗌 Pain 🗌 Fever 🗌 Asthenia (chronic fatigue) 🗌 Others (specify)…………..…………

- 1. What do you think it was due to?:
     - - - Symptoms very difficult to control despite medication
         - Lack of medical attention
         - Delays in medication administration
         - Others (specify) ……………………………
  2. Did you (family) have the support of professionals when you needed it?:

🗌 Always 🗌 Most of the time 🗌 Sometimes

🗌 Rarely 🗌 Never 🗌 Don’t know/Don’t answer

- 1. If you establish differences between doctors, nurses or other personnel, specify them:

…………………………………………………………………………………………………

- 1. Did you (family) receive medical information about the patient's situation / prognosis?

🗌 Always 🗌 Most of the time 🗌 Sometimes

🗌 Rarely 🗌 Never 🗌 Don’t know/Don’t answer

- 1. Were you (family) sufficiently informed of:

The resources available for patient care[[3]](#endnote-2): 🗌 YES 🗌 NO

Different options for treatment [[4]](#endnote-3): 🗌 YES 🗌 NO

The comfort measures that could be adopted [[5]](#endnote-4): 🗌 YES 🗌 NO

- 1. Did you (family) have the opportunity to discuss your concerns with the nurse and/or doctor?

🗌 Always 🗌 Most of the time 🗌 Sometimes

🗌 Rarely 🗌 Never 🗌 Don’t know/Don’t answer

- 1. Did you (family) know the severity of the disease before?

🗌 YES 🗌 NO

- 1. Did you (family) know it in the last month?

🗌 YES 🗌 NO

- 1. In your opinion, the priority objective that guided the actions of health professionals was:
- The cure of the disease
- Your relative's well-being
- Extend your family member's life as much as possible
- Others (specify)…………………………….
  1. Did this goal match yours?
- YES
- Partially (specify)………… …………………………………………….
- No (specify)………………………………………………………………….
  1. Was the possibility of transferring your family member to your home considered? 🗌 YES 🗌 NO
  2. If the answer is yes, did you (family) participate in the decision to transfer him/her home? 🗌 YES 🗌 NO
  3. Was the possibility of transferring your family member **to the hospital considered?** 🗌 YES 🗌 NO
  4. If the answer is yes, did you (family) participate in the decision to transfer him/he to the hospital?

🗌 YES 🗌 NO

- 1. Was the possibility of transferring your family member **to the Palliative Care Unit considered**?

🗌 YES 🗌 NO

- 1. If the answer is yes, did you (family) participate in the decision to transfer him/he to the Palliative Care Unit?

🗌 YES 🗌 NO

- 1. Was the possibility of a **non-resuscitation order** (not to perform resuscitation maneuvers) considered?

🗌 YES 🗌 NO

- 1. If the answer is yes, did you (family) participate in the decision of a **non-resuscitation order**?

🗌 YES 🗌 NO

- 1. Was the possibility of **Terminal Sedation** raised**?** 🗌 YES 🗌 NO
  2. If the answer is yes, did you (family) participate in the decision of **Terminal Sedation**? 🗌 YES 🗌 NO
  3. Was the possibility of **Limitation of Therapeutic Effort** raised (withdrawal of life support measures and other measures aimed at healing)? 🗌 YES 🗌 NO
  4. If the answer is yes, did you (family) participate in the decision of **Limitation of Therapeutic Effort**?

🗌 YES 🗌 NO

- 1. Was it necessary to make some kind of decision that is not covered in the previous questions?

🗌 YES 🗌 NO

Specify:____________________________________________________________

- 1. If the answer is yes, did you (family) participate in those decisions? 🗌 YES 🗌 NO
  2. Did you act as the legal representative of the patient in any of the decisions? 🗌 YES 🗌 NO

If yes, please specify in which situation …………………………………………………………

- 1. Were you able to accompany your sick family member as long as you wanted? 🗌 YES 🗌 NO
  2. Did he/she have a single room during hospitalization/nursing home? 🗌 Not applicable 🗌 YES 🗌 NO
  3. Did you act as the legal representative of the patient in any of the decisions? 🗌 YES 🗌 NO

If yes, please specify in which situation …………………………………………………………

- 1. In general, communication with the patient was:

🗌 Very easy

🗌 Easy

🗌 Difficult

🗌 Very difficult

🗌 Nonexistent

🗌 Don’t know/Don’t answer

1. **During the last month, do you consider your sick family member..**

🗌 YES

- 1. Did he/she know or was aware of the severity of his / her illness?

🗌 YES

🗌 NO

🗌 he / she / suspected it

🗌 Don’t know/Don’t answer

- 1. Did he/she make decisions about particular issues that concerned him/her?

🗌 YES

🗌 NO

🗌 He did not say anything about it

🗌 Don’t know/Don’t answer

- 1. Were his or her spiritual needs met?

🗌 YES

🗌 NO

🗌 He/She did not want it

🗌 Don’t know/Don’t answer

- 1. Was the non-resuscitation order discussed with the patient?

🗌 YES

🗌 NO

🗌 Don’t know/Don’t answer

- 1. Did he/she complete a written advance directive?

🗌 NO

🗌 Don’t know/Don’t answer

🗌 YES

- 1. Did he/she die in the place where he/she wanted to?

🗌 YES

🗌 NO

🗌 Don’t know/Don’t answer

- 1. How did he/she die?

🗌 Aware

 Sedated

🗌 Others (specify)………………………………………………….

- 1. Did he/she want to die like that?

🗌 YES

🗌 NO

🗌 Don’t know/Don’t answer

- 1. During the last days... Was he/she accompanied by the people he/she wanted to be with?

🗌 NO

🗌 Don’t know/Don’t answer

1. **Your opinion regarding end-of-life care**
   1. You would rate the death of your relative as:

🗌 Very good

🗌 Good

🗌 Average

🗌 Bad

🗌 Very bad

- 1. Why?

……………………………………………………………………………………………………………………………………………………………………………………………………………………………………………………………………………………………………………………………………………………………………………………………………………….

- 1. You would rate your experience as:

🗌 Very good

🗌 Good

🗌 Average

🗌 Bad

🗌 Very bad

- 1. Why?

……………………………………………………………………………………………………………………………………………………………………………………………………………………………………………………………………………………………………………………………………………………………………………………………………………….

…………………………………………………………………………………………………………………………………….

- 1. What needs did you raise that were not addressed or were not adequately addressed?

……………………………………………………………………………………………………………………………………………………………………………………………………………………………………………………………………………………………………………………………………………………………………………………………………………….

…………………………………………………………………………………………………………………………………….

- 1. In your opinion, what aspects of the lived process should be improved?

……………………………………………………………………………………………………………………………………………………………………………………………………………………………………………………………………………………………………………………………………………………………………………………………………………….

…………………………………………………………………………………………………………………………………….

**THANK YOU FOR YOUR PARTICIPATION**

1. CÓDIGO CENTRO**: HVN:** Virgen de las Nieves;  **HCS:** Clínico San Cecilio;  **HMT:** Motril;  **HML:** Melilla;  **HBA:** Baza;  **DSG:** Distrito Sanitario Granada [↑](#footnote-ref-2)
2. TIPO DE INSTRUMENTO: **HC**: Historia Clínica; **CP**: Cuestionario familiares; **CF:** Cuestionario familiares [↑](#footnote-ref-3)
3. Resources available in palliative care: Home care, hospitalization, Day hospital, Home hospitalization, Telephone care, Adaptation of the home, orthopedic material (articulated beds, shower chairs, etc.), Care by the social worker or liaison nurse, etc. [↑](#endnote-ref-2)
4. Treatment options: Medical treatment for curative purposes (includes chemotherapy and radiotherapy), surgical treatment for curative purposes, palliative treatment (may include chemotherapy, radiotherapy and palliative surgery), terminal sedation, etc. [↑](#endnote-ref-3)
5. Comfort measures: pharmacological measures for symptom control, withdrawal of medication not intended for symptom control, palliative treatment of pressure ulcers, mouth care, diet modification accordint to the patient's preferences, elimination care (urinary and faecal ), etc. [↑](#endnote-ref-4)
