# Supplementary material for: Experiences of family caregivers of patients with terminal disease and the quality of end-of-life care received: a mixed methods study
Source: PeerJ. 2020 Dec 14;8:e10516. doi: 10.7717/peerj.10516 (PMC7745673; doi:10.7717/peerj.10516)
Supplement: Supplemental Information 3 [file peerj-08-10516-s003.doc]

CUESTIONARIO FAMILIARES

CÓDIGO:

| _ _ _ | _ _ | _ _ _ |
| --- | --- | --- |
| CENTRO[[1]](#footnote-2) | DOCUMENTO[[2]](#footnote-3) | Nº CASO |

1. **Datos socio-demográficos del informante**
   1. Edad……………
   2. Sexo: 🗌 Hombre 🗌 Mujer
   3. Lugar de residencia…………………………
   4. Estado civil: 🗌 Soltero/a 🗌 Casado/a /Separado/a 🗌 Divorciado/a 🗌 Viudo/a
   5. Relación con el familiar fallecido:

🗌 Cónyuge 🗌 Hijo/a 🗌 Padre/madre 🗌 Hermano/a 🗌 Nieto/a 🗌 Otros (especificar)……………………….……

1. **Datos socio-demográficos del paciente**
   1. Edad……………
   2. Sexo: 🗌 Hombre 🗌 Mujer
   3. Lugar de residencia…………………………
   4. Estado civil: 🗌 Soltero/a 🗌 Casado/a /Separado/a 🗌 Divorciado/a 🗌 Viudo/a
   5. Enfermedad que padecía: ………………………………………………………………………………
   6. Tiempo de evolución de la enfermedad:

Días ......

Meses ……

Años ……

- 1. Tiempo transcurrido desde el fallecimiento:

Días ……

Meses ……

- 1. Lugar del fallecimiento:

🗌 Domicilio familiar 🗌 Hospital: 🗌 Sala de encamación 🗌 UCI

🗌 Urgencias 🗌 Cuidados Paliativos 🗌 Otros (especificar)…………………………..

1. **Datos referidos al último mes**
   1. Durante el último mes su familiar pasó la mayor parte del tiempo: 🗌 En casa 🗌 Hospitalizado 🗌 Residencia

**EN CASA / RESIDENCIA:**

- 1. Visitas a urgencias: número de visitas:…………

Duración………. Motivo……………………………………………………………………..…

Duración………. Motivo……………………………………………………………………..…

Duración………. Motivo……………………………………………………………………..…

Duración………. Motivo……………………………………………………………………..…

- 1. Ingresos hospitalarios: número de ingresos:…………

Duración………. Motivo……………………………………………………………………..…

Duración………. Motivo……………………………………………………………………..…

Duración………. Motivo……………………………………………………………………..…

- 1. Atención en domicilio por el equipo de AP

Visitas de seguimiento y control. Número de visitas……….

Visitas puntuales a demanda. Número de visitas……….

- 1. Atención en domicilio por el equipo de Cuidados Paliativos

Visitas de seguimiento y control. Número de visitas……….

Visitas puntuales a demanda. Número de visitas……….

- 1. Consulta telefónica con el equipo de cuidados paliativos.

nº de veces………. Motivos de consulta ……………………………………………………

………………………………………………………………………………………………………..

- 1. Visitas a Unidad de Cuidados Paliativos, nº y motivos

nº de veces………. Motivos de consulta ……………………………….……………………

**HOSPITALIZADO:**

- 1. Visitas de los profesionales de la Unidad de Cuidados Paliativos. Número de visitas:……..…

Motivo……………………………………………………………………..……..………………

Motivo……………………………………………………………………..……..………………

Motivo……………………………………………………………………..……..………………

Motivo……………………………………………………………………..……..………………

Motivo……………………………………………………………………..……..………………

- 1. Traslados a la UCI: número de traslados:…………

Motivo……………………………………………………………………..……..………………

Motivo……………………………………………………………………..……..………………

Motivo……………………………………………………………………..……..………………

- 1. Pruebas que recuerda que le realizaron
- Biopsias
- Radiografías
- Ecografías
- TACs
- Resonancias magnéticas
- Endoscopias
- Electrocardiogramas
- Analíticas de sangre
- Analíticas de orina
- Punciones
- Otras: ___________________
  1. Medidas terapéuticas que se le administraron

🗌 Quimioterapia/ radioterapia 🗌 Diálisis 🗌 Sonda nasogástrica 🗌 Alimentación parenteral

🗌 Sonda vesical 🗌 Sueros 🗌 Ventilación Mecanica 🗌 Otras (especificar)………………………

1. **Durante el último mes**
   1. Qué síntomas manifestó su familiar:

🗌 Disnea 🗌 Vómitos 🗌 Confusión 🗌 Debilidad/ Cansancio 🗌 Dolor

🗌 Fiebre 🗌 otros (especificar)…………..…………

- 1. En general, ¿**considera que los síntomas** que se presentaron estuvieron controlados?

🗌 Siempre 🗌 La mayor parte del tiempo 🗌 A veces

🗌 Muy pocas veces 🗌 Nunca 🗌 No sabe/No contesta

- 1. En general, ¿**considera que el dolor** estuvo controlado?

🗌 Siempre 🗌 La mayor parte del tiempo 🗌 A veces

🗌 Muy pocas veces 🗌 Nunca 🗌 No sabe/No contesta

- 1. De los síntomas que presentaba su familiar ¿Qué síntomas se controlaron **mejor** ?

🗌 Disnea 🗌 Vómitos 🗌 Confusión 🗌 Astenia (cansancio)

🗌 Debilidad 🗌 Dolor 🗌 Fiebre 🗌 otros (especificar)…………..…………

- 1. De los síntomas que presentaba su familiar ¿Qué síntomas se controlaron **peor**?

🗌 Disnea 🗌 Vómitos 🗌 Confusión 🗌 Astenia (cansancio)

🗌 Debilidad 🗌 Dolor 🗌 Fiebre 🗌 otros (especificar)…………..…………

- 1. ¿A qué cree que se debió?:
     - - - Síntomas muy difíciles de controlar a pesar de la medicación
         - Falta de atención médica
         - Demoras en la administración de medicamentos
         - Otros (especificar) ……………………………
  2. ¿Contó usted (familia) con el apoyo de los profesionales cuando lo necesitó?:

🗌 Siempre 🗌 La mayor parte del tiempo 🗌 A veces

🗌 Muy pocas veces 🗌 Nunca 🗌 No sabe/No contesta

- 1. Si establece diferencias entre médicos, enfermeros u otro personal, especificarlas:

…………………………………………………………………………………………………

- 1. ¿Recibió usted (familia) información médica sobre la situación/pronostico del enfermo?

🗌 Siempre 🗌 La mayor parte del tiempo 🗌 A veces

🗌 Muy pocas veces 🗌 Nunca 🗌 No sabe/No contesta

- 1. Le informaron a usted (familia) de manera suficiente de:

Los recursos disponibles para la atención del enfermo[[3]](#endnote-2): 🗌 SI 🗌 NO

Las diferentes opciones de tratamiento posibles[[4]](#endnote-3): 🗌 SI 🗌 NO

Las medidas de confort que se podían adoptar[[5]](#endnote-4): 🗌 SI 🗌 NO

- 1. ¿Tuvo usted (familia) ocasión de comentar sus preocupaciones con la enfermera y/o el médico?

🗌 Siempre 🗌 La mayor parte del tiempo 🗌 A veces

🗌 Muy pocas veces 🗌 Nunca 🗌 No sabe/No contesta

- 1. ¿Conocía usted (familia) la gravedad de la enfermedad con anterioridad?

🗌 SI 🗌 NO

- 1. ¿Lo supo usted (familia) durante el último mes?

🗌 SI 🗌 NO

- 1. En su opinión, el objetivo prioritario que guió las actuaciones de los profesionales sanitarios fue:
- La curación de la enfermedad
- El bienestar de su familiar
- Prolongar la vida de su familiar lo máximo posible
- otros (especificar)…………………………….
  1. ¿Coincidió este objetivo con el suyo?
- SI
- Parcialmente (especificar)………… …………………………………………….
- No. (especificar)………………………………………………………………….
  1. ¿Se planteó la posibilidad del **traslado de su familiar al domicilio**? 🗌 SI 🗌 NO
  2. En caso afirmativo de la pregunta anterior ¿Participó usted (familia) en la decisión del traslado

al domicilio? 🗌 SI 🗌 NO

- 1. ¿Se planteó la posibilidad del **traslado al hospital?** 🗌 SI 🗌 NO
  2. En caso afirmativo de la pregunta anterior ¿Participó usted (familia) en la decisión del traslado

al hospital? 🗌 SI 🗌 NO

- 1. ¿Se planteó la posibilidad del **traslado a una Unidad de Cuidados Paliativos**? 🗌 SI 🗌 NO
  2. En caso afirmativo de la pregunta anterior ¿Participó usted (familia) en la decisión del traslado a una Unidad de Cuidados Paliativos? 🗌 SI 🗌 NO
  3. ¿Se planteó la posibilidad de **Orden de no reanimación** (no realizar maniobras

de reanimación)? 🗌 SI 🗌 NO

- 1. En caso afirmativo de la pregunta anterior ¿Participó usted (familia) en la decisión de la

orden de No Reanimación? 🗌 SI 🗌 NO

- 1. ¿Se planteó la posibilidad de **SedaciónTerminal?** 🗌 SI 🗌 NO
  2. En caso afirmativo de la pregunta anterior ¿Participó usted (familia) en la decisión de

la Sedación Terminal? 🗌 SI 🗌 NO

- 1. ¿Se planteó la posibilidad de **Limitación del Esfuerzo Terapéutico** (retirada de medidas de soporte vital y otras medidas destinadas a la curación)? 🗌 SI 🗌 NO
  2. En caso afirmativo de la pregunta anterior ¿Participó usted (familia) en la decisión de la Limitación del Esfuerzo Terapéutico? 🗌 SI 🗌 NO
  3. ¿Se tuvo que tomar algún tipo de decisión que **no esté contemplada en las preguntas anteriores**?

🗌 SI 🗌 NO

Especificar :____________________________________________________________

- 1. En caso afirmativo de la pregunta anterior ¿Participó usted (familia) en esas decisiones? 🗌 SI 🗌 NO
  2. ¿Actuó como representante legal del enfermo en alguna de las decisiones? 🗌 SI 🗌 NO

En caso afirmativo, especifique en qué situación …………………………………………………………

- 1. ¿Pudo acompañar todo el tiempo que usted quiso a su familiar enfermo? 🗌 SI 🗌 NO
  2. ¿Dispuso de habitación individual durante la hospitalización/residencia? 🗌 No procede 🗌 SI 🗌 NO
  3. ¿Actuó como representante legal del enfermo en alguna de las decisiones? 🗌 SI 🗌 NO

En caso afirmativo, especifique en qué situación …………………………………………………………

- 1. La comunicación con el enfermo ha sido en general:

🗌 Muy fácil

🗌 Fácil

🗌 Difícil

🗌 Muy difícil

🗌 Nula

🗌 No sabe/No contesta

1. **Durante el último mes, considera que su familiar enfermo**

🗌 SI

- 1. ¿Conocía o estaba informado de la gravedad de su enfermedad?

🗌 SI

🗌 NO

🗌 Lo sospechaba

🗌 No sabe/No contesta

- 1. ¿Tomó decisiones sobre asuntos particulares que le preocupaban?

🗌 SI

🗌 NO

🗌 No manifestó nada al respecto

🗌 No sabe/No contesta

- 1. ¿Se atendieron sus necesidades espirituales?

🗌 SI

🗌 NO

🗌 No quiso

🗌 No sabe/No contesta

- 1. ¿Se le planteó al enfermo la orden de no reanimación?

🗌 SI

🗌 NO

🗌 No sabe/No contesta

- 1. ¿Disponía de voluntades anticipadas?

🗌 NO

🗌 No sabe/No contesta

🗌 SI

- 1. ¿Murió en el lugar donde quería morir?

🗌 SI

🗌 NO

🗌 No sabe/No contesta

- 1. ¿En qué estado murió?

🗌 Consciente

🗌 Sedado

🗌 Otros (especificar)………………………………………………….

- 1. ¿Y él o ella quería morir en ese estado?

🗌 SI

🗌 NO

🗌 No sabe/No contesta

- 1. En los últimos días… ¿Estuvo acompañado de las personas con las que deseaba estar?

🗌 NO

🗌 No sabe/No contesta

1. **Su opinión sobre la atención en el proceso final de la vida**
   1. Calificaría la muerte de su familiar como:

🗌 Muy buena

🗌 Buena

🗌 Regular

🗌 Mala

🗌 Muy mala

- 1. ¿Por qué?

……………………………………………………………………………………………………………………………………………………………………………………………………………………………………………………………………………………………………………………………………………………………………………………………………………….

- 1. Calificaría su experiencia como:

🗌 Muy buena

🗌 Buena

🗌 Regular

🗌 Mala

🗌 Muy mala

- 1. ¿Por qué?

……………………………………………………………………………………………………………………………………………………………………………………………………………………………………………………………………………………………………………………………………………………………………………………………………………….

…………………………………………………………………………………………………………………………………….

- 1. ¿Qué necesidades de las que ustedes plantearon no fueron atendidas o no lo fueron de manera adecuada?

……………………………………………………………………………………………………………………………………………………………………………………………………………………………………………………………………………………………………………………………………………………………………………………………………………….

…………………………………………………………………………………………………………………………………….

- 1. En su opinión, ¿qué aspectos del proceso vivido deben mejorar?

……………………………………………………………………………………………………………………………………………………………………………………………………………………………………………………………………………………………………………………………………………………………………………………………………………….

…………………………………………………………………………………………………………………………………….

**MUCHAS GRACIAS POR SU PARTICIPACIÓN**

1. CÓDIGO CENTRO**: HVN:** Virgen de las Nieves;  **HCS:** Clínico San Cecilio;  **HMT:** Motril;  **HML:** Melilla;  **HBA:** Baza;  **DSG:** Distrito Sanitario Granada [↑](#footnote-ref-2)
2. TIPO DE INSTRUMENTO: **HC**: Historia Clínica; **CP**: Cuestionario familiares; **CF:** Cuestionario familiares [↑](#footnote-ref-3)
3. Recursos disponibles en cuidados paliativos: Atención domiciliaria, hospitalización, Hospital de día, Hospitalización domiciliaria, Atención telefónica, Adaptación del domicilio, material ortopédico (camas articuladas, sillas de ducha, etc.), Atención por la trabajadora social o enfermera de enlace, etc. [↑](#endnote-ref-2)
4. Opciones de tratamiento: Tratamiento médico con fines curativos (incluye quimioterapia y radioterapia), tratamiento quirúrgico con fines curativos, tratamiento paliativo (puede incluir quimioterapia, radioterapia y cirugía paliativa), sedación terminal, etc. [↑](#endnote-ref-3)
5. Medidas de confort: medidas farmacológicas para control de síntomas, retirada de medicación no destinada a control de síntomas, tratamiento paliativo de úlceras por presión, cuidados de la boca, modificación de la dieta a gusto del paciente, cuidados de la eliminación (urinaria y fecal), etc. [↑](#endnote-ref-4)
